# 2,3,5,4′-Tetrahydroxystilbene-2-O-beta-D-glucoside Reverses Stress-Induced Depression via Inflammatory and Oxidative Stress Pathways

**DOI:** 10.1155/2018/9501427

**Published:** 2018-09-19

**Authors:** Cheng-Yong Jiang, Xiao-Yan Qin, Mi-Mi Yuan, Gui-Jiang Lu, Yong Cheng

**Affiliations:** Key Laboratory of Ethnomedicine for Ministry of Education, Center on Translational Neuroscience, College of Life and Environmental Sciences, Minzu University of China, Beijing 100081, China

## Abstract

Major depressive disorder (MDD) is a chronic mental disease that adversely affects human mood and cognition. Many first-line antidepressant drugs have high rates of partial responsiveness or nonresponsiveness with side effects, and finding more effective drugs for the treatment of depression is therefore urgently needed. THSG, a main active compound of the traditional Chinese herb *Polygonum multiflorum*, reportedly acts as a neuroprotective agent. This study aimed to illustrate whether THSG prevents depressive-like behaviors induced by chronic restraint stress (CRS) in an MDD mouse model. Our results demonstrated that the peripheral administration of different THSG doses (10 mg/kg, 20 mg/kg, and 40 mg/kg) reversed the depressive-like behaviors in CRS mice as measured by the tail suspension test, forced swimming test, and open-field test. Further analyses suggested that THSG treatment reduced oxidative stress in both the central and peripheral nervous systems of CRS mice. In addition, heightened inflammatory responses, demonstrated by the increased expression of proinflammatory factors (TNF-*α*, IL-1*β*, and IL-6), in hippocampal and prefrontal cortex tissues of CRS mice were inhibited by THSG administration. THSG also restored the diminished Akt signaling pathway in the brains of CRS mice. Moreover, our data suggest increased astrocyte proliferation and neurogenesis in the hippocampus of CRS mice after THSG treatment. Taken together, our results demonstrated an antidepressant effect of THSG in a mouse model of MDD for the first time, and oxidative stress and inflammatory pathways were determined to play roles in this effect.

## 1. Introduction

Mental illnesses, such as major depressive disorder (MDD), Parkinson's disease (PD), and Alzheimer disease (AD), are global public health problems [1] that immensely threaten the life qualities of patients. As a type of affective disorder, MDD is a comprehensive mental illness characterized by depression, mental retardation, and language reduction. MDD patients often suffer from some somatic maladaptation symptoms, including loss of appetite, decreased activity, decreased interest, fatigue, sleep disorders, and even suicidal behavior. According to the World Health Organization (WHO) estimation, 16% of the global population is influenced by MDD to varying degrees [2]. Although MDD places serious mental and economic burdens on society, its pathogenesis has not been elucidated until now [3]. Many first-line antidepressant drugs have high rates of partial responsiveness or nonresponsiveness [4], induce delayed and partial side effects [5], and cause remission and residual symptomatology in affected patients, often leading to a high rate of recurrence. Therefore, exploring the pathogenesis of MDD and searching for efficient antidepressant drugs is extraordinarily significant for clinical treatment.

Numerous studies have demonstrated that stress-induced mental issues and stress-induced pathological lesions are the two main etiologies of depression [6] that occur in encephalic regions, particularly in prefrontal cortex (PFC) and hippocampal tissues [7]. Preclinical research has demonstrated many pathological symptoms in MDD patients, including deficiencies of neurogenesis, inflammatory responses, oxidative stress, and abnormal cytokine secretion [8]. A physical stress-induced MDD animal model with behavioral and cognitive deficits not only shows a reduced number of proliferating hippocampal progenitor cells [9, 10] but also shows a notably decreased number of microglia in PFC and hippocampal tissues [11, 12]. Microglia, linked to coordinating neural network interactions among neurons [13], were stimulated and shown to represent an important component of neuroinflammation [14]. In a meta-analysis, psychological stress was shown to promote acute proinflammatory responses with elevations in interleukin (IL)-6, IL-1*β*, and tumor necrosis factor alpha (TNF-*α*) levels [15]. Additionally, proinflammatory cytokines are involved in the generation of reactive oxygen species (ROS) produced by impaired cells [16]. As a consequence, oxidative stress arises from an imbalance between oxidant and antioxidant compounds in favor of excessive free radical generation. Excessive ROS production can induce cellular damage by enzyme inactivation, lipid peroxidation, protein damage, and DNA modification [17], ultimately leading to necrosis or apoptosis. For example, in hippocampal tissues of MDD patients, the rates of neuronal proliferation and survival are both reduced [18, 19], while a high apoptosis rate is observable. As noted, the Akt and Erk signaling pathways function in neuronal apoptosis by activating the expression of downstream genes [20, 21] and may thus represent promising therapeutic targets for depression.

Herbal therapies have been used for many years, and the curative effects of medicinal plants, which have lower economic costs and fewer side effects, have been proven to treat many clinical diseases [21]. 2,3,5,4′-Tetrahydroxystilbene-2-O-beta-D-glucoside (THSG) is one of the most effective ingredients in the traditional Chinese edible herb *Polygonum multiflorum* (PM) [22]. Pharmacological research conducted via modern Western medicine has proven that PM has antioxidant and antiaging properties and that it enhances immunity, regulates blood lipids, prevents atherosclerosis, and improves learning and memory ability [23, 24]. THSG was demonstrated to enhance the activity of superoxide dismutase (SOD) and reduce the malondialdehyde (MDA) content in patients with colitis, which suggests that THSG functions as an antioxidant to eliminate free radicals [25]. Furthermore, THSG can also increase the density of dendritic spines in the CA1 area of the hippocampus and increase the synaptic plasticity of the hippocampus [26]. Whether THSG exerts an antidepressant effect, however, has not been studied. In this study, we investigated the antidepressant-like effects of THSG using a well-established animal depression mouse model of chronic restraint stress (CRS). In our study, we detected the depressant-like behaviors of the model mice via classical behavior tests, including the tail suspension test (TST), forced swimming test (FST), and open-field test (OFT). To further investigate how THSG exerts its antidepressive effects, oxidative stress-related markers (MDA, GSH-PX, and T-AOC) and inflammatory markers (TNF-*α*, IL-1*β*, IL-6, and NOS-2) were assayed using biochemistry technology. The key protein factors in the AKT and ERK signaling pathways p-Akt, total Akt, total Erk, and p-Erk were assessed to explore the potential molecular mechanisms underlying the neuroprotective effects of THSG in CRS mice.

## 2. Material and Methods

### 2.1. Experimental Animals

One-month-old male C57BL/6 mice were purchased from Beijing Vital River Laboratory Animal Technology Co. Ltd. Upon arrival, all experimental mice were given food and water in a temperature- and humidity-controlled room with a light : dark cycle of 12 h. All animal experiments and protocols were approved by the Animal Care and Use Committee of Minzu University of China.

### 2.2. CRS Procedures and Experimental Design

**Figure 1:**
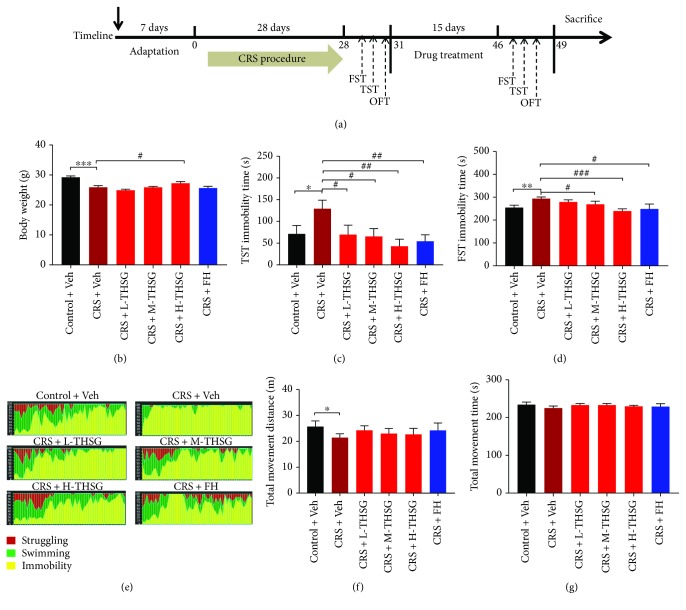
THSG alleviated depression-related behaviors in CRS-induced model mice. Model mice were exposed to CRS for 4 weeks according to the timeline (a), and different concentrations of THSG were administered for 2 weeks. The behavior tests were carried out after THSG (10 mg/ml, 20 mg/ml, and 40 mg/ml), vehicle, and fluoxetine (10 mg/ml) treatment. The changes in mouse body weights are shown in (b). The immobility times in the TST and FST are shown in (c, d, e). The distances and times of mouse locomotor activity in the OFT are shown in (f, g). Values are expressed as the mean ± SEM, with 10 mice in the control + Veh group and 9 mice in the other groups. For statistical significance, ^∗^*p* < 0.05, ^∗∗^*p* < 0.01, and ^∗∗∗^*p* < 0.001 versus the control + Veh group; ^#^*p* < 0.05, ^##^*p* < 0.01, and ^###^*p* < 0.001 versus the CRS + Veh group.

To induce depressive-like behaviors in mice, the mice were subjected to CRS according to a previous study [27] with modifications. Briefly, the mice were restrained in cylinder-shaped tubes from 9 a.m. to 4 p.m. every day for 28 days. After 28 days of CRS, the mice were weighed and subjected to the FST, TST, and OFT to assess their depressive-like behaviors. The mice were then subcutaneously injected with a vehicle or THSG (purchased from Sheng Haiyuan Leaf Biotechnology Co. Ltd.) for 15 days to evaluate whether THSG exerts antidepressant effects. In addition, one group of CRS mice was intragastrically administered 10 mg/kg fluoxetine (FH) for 15 days as a positive control. All mice were treated every other day, and all drugs were administered at the same volume (0.2 ml) but at different concentrations. After 15 days of drug treatment, the mice were subjected to behavioral tests and then sacrificed; their blood and brain tissues were collected for biochemical assays. The experimental procedure is shown in Figure 1(a).

### 2.3. Behavior Assays for Major Depressive Disorder

#### 2.3.1. Force Swimming Test (FST)

The FST is an effective method for examining the behavioral desperation of depressed mice and is used to measure the effect of antidepressant medicine treatment. According to a previous study [28], mice were individually forced to swim in a cylindrical plexiglass container (40 cm in height, 15 cm in diameter) filled with 2 l of water (approximately 25°C). The foot and tail of the mouse were confirmed to not touch the bottom of the container throughout the 6 min of swimming. After the first 1 min of adaption, the immobility time of the mouse in the last 5 min was recorded and analyzed by a forced swimming analysis system (Chengdu Taimeng Software Co. Ltd., Sichuan). The mice were deemed immobile when they floated in water without struggling and exerted slight movements to maintain body balance.

#### 2.3.2. Tail Suspension Test (TST)

The TST is another model used to express the learning helplessness phenomenon in MDD model mice. To assess the antidepressant effect of THSG, the TST was carried out as described by Willner [29] with minor improvements. Briefly, the mouse was individually suspended by gluing approximately 1 cm of the tip of the tail to a fixed metal rod using adhesive tape, ensuring that the distance between the mouse head and floor was at least 10 cm. The mouse was suspended for 6 mins, and the last 5 min of immobility time was accounted and analyzed by a tail suspension analysis system (Chengdu Taimeng Software Co. Ltd.). Mice were deemed immobile when they stopped struggling and became completely motionless.

#### 2.3.3. Open-Field Test (OFT)

The OFT is used to evaluate the autonomous behavior, inquiry behavior, and tension of experimental animals in unfamiliar environments. To measure the antidepressant effect of THSG, the OFT was performed in accordance with a previously described method [30, 31]. The open-field apparatus (50 cm × 50 cm × 45 cm) consisted of 25 white square grids (10 cm × 10 cm for each grid), and the open field was enclosed with a black metal plate. Mice were individually placed onto the central grid of the floor and allowed to freely explore for 6 min under a fluorescent lamp. The mobility states of the mice in the open field were recorded and analyzed by an open-field system (Chengdu Taimeng Software Co. Ltd., Sichuan). When one mouse finished the test, the floor of the open square was wiped with 75% alcohol and naturally volatilized before the next mouse used the system.

### 2.4. Detection of Oxidation-Associated Markers

#### 2.4.1. Determination of MDA, GSH-PX, and T-AOC in Serum

Peripheral serum samples (1.0 ml) were collected from the retroorbital vessels of the mice and then clotted for 2 h at 4°C. After 2 h, the mouse blood was centrifuged at 4000 rpm/min for 15 min at 4°C. The upper serum layers (200 *μ*l) were separated, collected into new Eppendorf (EP) tubes, and stored at −80°C until use.

To determine whether oxidative stress exists in MDD mice, MDA, glutathione peroxidase (GSH-PX), and total antioxidant capacity (T-AOC) were detected by chemical colorimetric analyses. (1) The MDA levels were analyzed using the thiobarbituric acid (TBA) method by an MDA kit (Nanjing Jiancheng Bioengineering Institute, Nanjing, China) according to the manufacturer's recommendations. MDA in serum reacted with the TBA-generated MDA-TBA adduct. The adduct was maximally absorbed at a wavelength of 532 nm, which was monitored using a microtiter plate reader (Perkin Elmer Enterprise Management Co. Ltd., Shanghai). The MDA content in serum was expressed as micromoles per liter (*μ*mol/l). (2) GSH-PX activity was assessed by a glutathione peroxidase assay kit (Nanjing Jiancheng Bioengineering Institute). The absorbance of stable yellow reaction products was measured at 412 nm using a microtiter plate reader. The GSH-PX activity in serum was expressed as units per ml of plasma (U/ml). (3) The T-AOC content was determined using a commercial reagent kit (Nanjing Jiancheng Bioengineering Institute) according to the manufacturer's instructions, and the T-AOC activity was expressed as units per ml of plasma (U/ml).

#### 2.4.2. Determination of MDA, GSH-PX, and T-AOC in the Hippocampus and Prefrontal Cortex

Mice were sacrificed by cervical dislocation, and their hippocampal and PFC tissues were immediately dissected from the left and right brains at 4°C and placed in PBS buffer (0.01 M). The hippocampal and PFC tissues were separately placed into EP tubes and stored at −80°C until use in the biomarker assays. The hippocampal and PFC samples were lysed and homogenized using tissue lysis buffer (containing protease and phosphatase inhibitors) in an ice bath for at least 30 min. Next, the total homogenate was centrifuged at 13,000 rpm for 15 min at 4°C. The supernatants were collected, divided into new tubes, and stored at −80°C for use in Western blot and oxidation-associated biomarker assays.

The total protein concentration in each sample was quantified using a bicinchoninic acid (BCA) assay kit (Shanghai Biyuntian Biotechnology Co. Ltd.) based on a standard curve. The MDA, GSH-PX, and T-AOC contents were determined using MDA, GSH-PX, and T-AOC kits, respectively, according to the manufacturer's recommendations with a slight modification in dosage. The MDA content was expressed as nmol per milligram of protein (nmol/mg), and the GSH-PX and T-AOC activities were expressed as units per milligram of protein. Apart from these methods, the other protocols utilized were basically similar to those described above for serum detection.

### 2.5. Quantitative Real-Time PCR

Total RNAs were extracted from hippocampal and PFC tissues using an mRNA isolation kit (Beijing Zhuangmeng International Biological Gene Technology Co. Ltd.) and stored at −80°C prior to use in the qRT-PCR assay. All steps were performed according to the manufacturer's instructions. Briefly, hippocampal and PFC tissues in 1.5 ml EP tubes were homogenized with 1 ml of TRIzol TM reagent for at least 3 min. The lysates were incubated at room temperature for 15–30 min and then centrifuged (12,000 rpm for 10 min at 4°C). The colorless upper liquid was collected into a new RNase-free tube, and 0.2 ml of chloroform was added. After 15 s of severe oscillation and 3 min of incubation at room temperature, the samples were centrifuged into three layers (lower red phenol-chloroform phase, interphase, and a colorless upper aqueous phase). The colorless upper aqueous phase was collected into an RNase-free adsorption column set in the collection tube, the solution was centrifuged (12,000 rpm for 45 s at 4°C), and 0.5 ml of isopropanol was added. The aqueous phase was removed from the collection tube, and the operation was repeated twice more, centrifuging at 13,000 rpm for 2 min at 4°C the final time. The adsorption column containing RNA was placed into a new RNase-free collection tube, 40–100 *μ*l of RNase-free water was added, and the mixture was centrifuged (12,000 rpm for 1 min at 4°C). The harvested RNA samples in the collection tubes were subjected to absorbance measurements at 260 nm and 280 nm using the Fast Thermal Cycler A300. RNA with an OD260/OD280 ratio of 1.8–2 was reverse-transcribed into cDNA using Prime Script™ RT Master Mix following the manufacturer's instructions. Briefly, the reaction system comprised a total volume of 20 *μ*l, including 3 *μ*l of the RT Enzyme Mix, 7 *μ*l of the RT Reaction Mix, and 10 *μ*l of the RNA sample. The blending mix was incubated at 40°C for 30 min and at 85°C for 5 min. The template DNA was subjected to qRT-PCR on a LightCycler^®^ 96 system. The 25 *μ*l reaction system included 12.5 *μ*l of SYBR Green I SuperMix, 1 *μ*l of both the sense and antisense primers, 2 *μ*l of template DNA, and 8.5 *μ*l of RNase-free water. The cycling conditions were as follows: preincubation at 95°C for 180 s, followed by 40 cycles of synthesis at 95°C for 10 s and 56°C for 30 s and melting at 95°C for 10 s, 65°C for 60 s, and 97°C for 1 s. All primers were designed by Kingsley Biotechnology Co. Ltd. (Shanghai). The primer sequences were as follows: *β*-actin (5′-AGACCTCTATGCCAACACAGT-3′, 3′-TCCTGCTTGCTGATCCACAT-5′); TNF-*α* (5′-CGTGGAACTGGCAGAAGAG-3′, 3′-TGAGAAGAGGCTGAGACATA-5′); NOS-2 (5′-CGAGACGGATAGGCAGAGATT-3′, 3′-GGAGGAGCTGATGGAGTAGTA-5′); IL-1*β* (5′-ATCTCGCAGCAGCACATCA-3′, 3′-CCAGCAGGTTATCATCATCATCC-5′); and IL-6 (5′-TTCCATCCAGTTGCCTTCTTG-3′, 3′-AAGCCTCCGACTTGTGAA-5′). The relative mRNA expression levels were analyzed using the 2^−ΔΔCt^ method.

### 2.6. Western Blot Analysis

As previously reported [32, 33], total proteins in the lysate samples were adjusted to equal concentrations, ensuring that equal amounts of protein were mixed with loading buffer (P0015, Blue Sky Biotechnology Co. Ltd.) and loaded equally (15 *μ*l) onto each lane. The proteins were separated by 10% SDS-PAGE for 1.5 h and then transferred to polyvinylidene fluoride (PVDF, Millipore) membranes using the semidry transmembrane method. The membrane was blocked with 5% (*w*/*v*) skim milk in Tris-buffered saline (TBST) for 1 h at room temperature. After washing with TBST 2 times for 10 min each, the membrane was incubated with a primary antibody at 4°C overnight with slight rocking. After washing with TBST 3 times for 10 min each, the membrane was incubated with horseradish peroxidase- (HRP-) conjugated secondary antibodies (Jackson ImmunoResearch Laboratories Inc., PA) at room temperature for 1 h. The membrane was incubated with developing liquid for 1 min after washing 3 times for 10 min each. The signal was visualized by a chemiluminescence imager (Beijing Yuanping Hao Biotechnology Co. Ltd.), and protein bands were quantified using the ImageJ software. All primary antibodies used, including *β*-actin (Sigma), total Akt (Cell Signaling Technology), p-Akt (Cell Signaling Technology), total Erk (Cell Signaling Technology), p-Erk (Cell Signaling Technology), GFAP (Cell Signaling Technology), and doublecortin (DCX, Cell Signaling Technology), were diluted according to the products' specifications. The level of *β*-actin was used as the internal control.

### 2.7. Data Analysis

All data analyses were conducted using the commercially available GraphPad Prism 5.0 software. Results are expressed as the mean ± SEM (standard error of the mean). Significance among groups was calculated using one-way analysis of variance (ANOVA) followed by unpaired independent sample *t*-tests. *p* values less than 0.05 (*p* < 0.05) were considered statistically significant.

## 3. Results

### 3.1. Effect of THSG Treatment on Behavior Parameters

#### 3.1.1. Effect of THSG Treatment on Mouse Body Weight

As showed in Figure 1(b), the body weights of the CRS + Veh mice were significantly decreased compared with those of the vehicle-treated mice (*p* < 0.05), which was similar to previous reports [34, 35]. When treated with a different concentration of THSG, the body weights slightly recovered compared with those of CRS + Veh mice. However, the differences between the body weights of mice treated with CRS + Veh and low-dose THSG (L-THSG, 10 mg/kg) or medium-dose THSG (M-THSG, 20 mg/kg) were not significant (*p* > 0.05); high-dose THSG (H-THSG, 40 mg/kg) treatment significantly reversed the body weight changes (*p* < 0.05).

#### 3.1.2. Effect of THSG Treatment on Immobility Times in the FST and TST

The TST and FST were performed to evaluate the behavior disparities of the tested mice. Figures 1(c)–1(e) show that CRS exposure reduced the immobility times of mice in the TST and FST compared with those of vehicle-treated mice, and ANOVA analysis confirmed a significant difference (*p* < 0.01). A low THSG concentration (10 mg/kg) significantly reversed the behavior change of MDD mice in the TST, but this change was not significant in the FST. However, both medium and high THSG concentrations (20 mg/kg and 40 mg/kg) significantly reduced the immobility times of mice exposed to CRS in the TST and FST, indicating THSG exerted this antidepressant effect in a dose-dependent manner. The antidepressant effect of THSG (40 mg/kg) was similar or even better than that of a prescribed antidepressant FH, suggesting the efficacy of THSG as a potential antidepressant.

#### 3.1.3. Effect of THSG Treatment on OFT Performance

To estimate the effect of THSG treatment on the locomotor activity of mice, an OPT was carried out.  Figure 1(f) illustrates that CRS markedly decreased the total distances that the mice moved (*p* < 0.05). However, compared with the distances travelled by CRS-Veh group mice, THSG-treated mice did not show significantly increased movement distances (*p* > 0.05). Furthermore, no significant differences in mouse movement times were observed among the 6 groups (*p* > 0.05), as shown in Figure 1(g). This result was similar to that in a previous report [36], which suggests that MDD mice may not sufficiently represent significant changes in locomotor activity, and the effects of THSG on the TST and FST were not caused by the locomotive activity.

### 3.2. THSG Ameliorates CRS-Induced Oxidative Stress In Vivo

#### 3.2.1. THSG Reduced MDA Level in CRS Mouse Cell

The increased MDA content can be used as an oxidative stress-induced damage marker [37]. Mice exposed to CRS displayed notably enhanced MDA contents in both peripheral serum (Table 1) and the hippocampus (23.50 ± 1.803 versus 18.86 ± 1.747; 0.6516 ± 0.041 versus 0.7906 ± 0.027; *p* < 0.05), while mice treated with THSG (10 mg/kg, 20 mg/kg, and 40 mg/kg) exhibited significantly reduced MDA contents in both peripheral serum and the hippocampus (*p* < 0.001, 0.01, or 0.05). Although a similar change in MDA content was also observed in the PFC, significant differences were not detected among the groups. Furthermore, reversal of the effect with FH (10 mg/ml) was similar with THSG (40 mg/ml).

#### 3.2.2. Effect of THSG Treatment on GSH-PX Activity

GSH-PX is known as one of the most important antioxidative stress enzymes. Significantly elevated GSH-PX activities were observed in peripheral serum (Table 1) and PFC tissues (1089 ± 31.64 versus 905.1 ± 64.37; 320.3 ± 54.46 versus 215.2 ± 15.37, *p* < 0.01 or 0.05) of CRS mice, but the changes were gradually suppressed as different concentrations of THSG were administered. However, compared with that in vehicle control mice, the GSH-PX activity in hippocampal tissue from CRS + Veh mice was reduced (143.3 ± 6.73 versus 169.1 ± 8.45, *p* < 0.05). Moreover, THSG (10 mg/kg, 20 mg/kg, and 40 mg/kg) treatment promoted GSH-PX activity in a dose-dependent manner, as this effect was significant at dose of 40 mg/ml (*p* < 0.05) but not at low or medium doses. These results suggest that THSG restored homeostasis in the mice after stress.

#### 3.2.3. Effect of THSG Treatment on T-AOC Activity

As demonstrated in Table 1, the T-AOC activities in peripheral serum and hippocampal tissues from CRS + Veh group mice were significantly enhanced (6.11 ± 0.44 versus 5.12 ± 1.05; 0.258 ± 0.09 versus 0.188 ± 0.039, *p* < 0.05), and no significant T-AOC activity was observed in PFC tissues (0.26 ± 0.046 versus 0.24 ± 0.047, *p* > 0.05). After THSG treatment, the effect of CRS exposure was suppressed. Meanwhile, markedly reduced T-AOC activity was observed at a medium dose (20 mg/ml, *p* < 0.01 or *p* < 0.05) rather than a high dose (40 mg/ml, *p* > 0.05) in the hippocampus and PFC. The above results indicate that oxidative stress may play an important role in depressant-like behavior [37–39], and THSG possibly exerts antidepressant effects in this manner.

### 3.3. Effect of THSG on Inflammatory Cytokines

**Figure 2:**
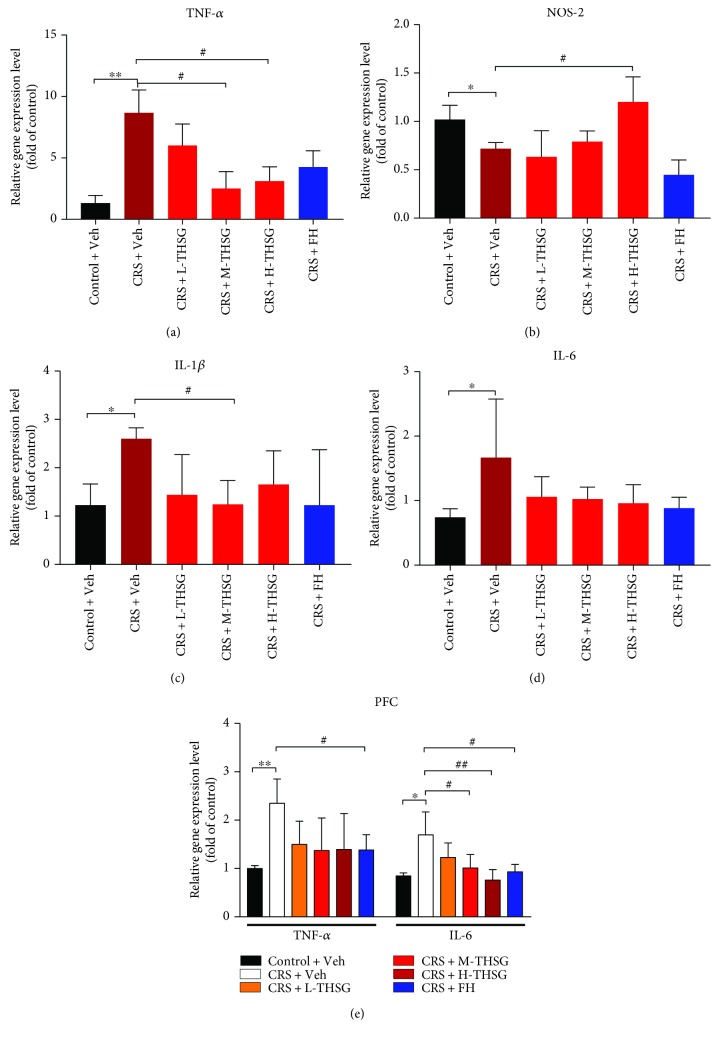
Effect of THSG on anti-inflammatory properties in the hippocampus and prefrontal cortex. Total TNF-*α*, NOS-2, IL-1*β*, and IL-6 mRNAs were isolated from the hippocampal and prefrontal cortex tissues using an mRNA isolation kit. The mRNA expression levels of TNF-*α*, NOS-2, IL-1*β*, and IL-6 in the mouse hippocampi are presented in (a, b, c, d). Group (e) shows the mRNA expression levels of TNF-*α* and IL-6 in mouse prefrontal cortex tissue. Values are expressed as the mean ± SEM (*n* = 3). For statistical significance, ^∗^*p* < 0.05 and ^∗∗^*p* < 0.01 versus the control + Veh group; ^#^*p* < 0.05 and ^##^*p* < 0.01 versus the CRS + Veh group.

The effects of THSG on inflammatory cytokine are presented in Figure 2. Figures 2(a), 2(c), and 2(d) show that CRS exposure significantly elevated the mRNA expression levels of TNF-*α*, IL-1*β*, and IL-6 in the mouse hippocampus compared with that in the control + Veh group (*p* < 0.01 or *p* < 0.05). Medium (20 mg/kg) and high (40 mg/kg) doses of THSG markedly decreased the enhancement of TNF-*α* and IL-6 levels in hippocampal tissue induced by CRS (*p* < 0.05). However, only the medium THSG dose (20 mg/kg) significantly reduced IL-1*β* gene expression in the hippocampus (*p* < 0.05). By contrast, NOS-2 gene expression in the mouse hippocampus was significantly reduced by CRS exposure, as shown in Figure 1(b) (*p* < 0.05). H-THSG treatment (40 mg/kg) markedly reversed the change in NOS-2 mRNA expression (*p* < 0.05). Both low and high doses of THSG (10 mg/kg, 40 mg/kg) increased NOS-2 mRNA expression, but the differences between these groups and the CRS + Veh were not significant (*p* > 0.05). Moreover, mice administered 10 mg/kg FH were not significantly different from those of the CRS + Veh group (*p* > 0.05), which suggested that THSG possibly exerts anti-inflammatory effects more vigorously than FH.

Changes in the PFC TNF-*α* and IL-6 mRNA expression levels among the groups are shown in Figure 2(e). The PFC expression levels of TNF-*α* and IL-6 were significantly increased in CRS mice compared with those in the vehicle-treated group (*p* < 0.01 or *p* < 0.05). Medium and high doses of THSG (20 mg/kg, 40 mg/kg) significantly restored the changes in IL-6 expression (*p* < 0.01 or *p* < 0.05) in a dose-dependent manner. However, no significant difference in changes in the TNF-*α* mRNA expression level was observed in the THSG-treated mice (*p* > 0.05). Notably, in PFC tissue, FH treatment reduced both the TNF-*α* and IL-6 mRNA expression levels significantly compared with those in the CRS + Veh group (*p* < 0.05). The above results illustrated that CRS exposure activated inflammatory reactions in the mouse central nervous system (CNS), and as the depression symptoms eased, the inflammatory cytokines gradually recovered to normal levels.

### 3.4. Effect of THSG Treatment on the Akt Pathway in the CNS

**Figure 3:**
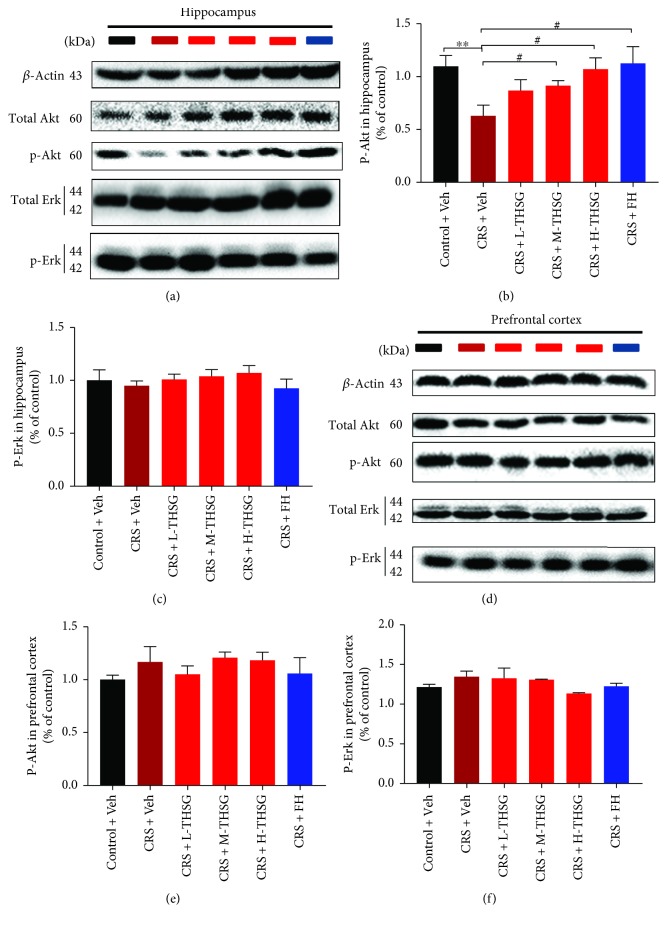
P-Akt plays an important role in the antidepressant effect of THSG. Tissue lysates were subjected to Western blot analysis. Bands corresponding to the p-Akt and p-Erk proteins in the hippocampus are shown in (a). The relative band densities of p-Akt and p-Erk in the hippocampus are shown in (b, c). Bands corresponding to the p-Akt and p-Erk proteins in the PFC are shown in (d). The relative band densities of p-Akt and p-Erk in the hippocampus are shown in (e, f). The protein band densities were analyzed using the ImageJ software. Values are expressed as the mean ± SEM (*n* = 4). For statistical significance, ^∗∗^*p* < 0.01 versus the control + Veh group; ^#^*p* < 0.05 versus the CRS + Veh group.

To further explore the probable molecular mechanism underlying THSG antidepressant activity, our study focused on two neuroprotective signaling pathways, p-Akt and p-Erk. Western blot analyses of p-Akt and p-Erk in the hippocampus and PFC are shown in Figure 3. Hippocampal p-Akt protein expression was notably downregulated (*p* < 0.01) in mice exposed in CRS stress, as shown in Figures 3(a) and 3(b), while THSG treatment abolished this alteration. However, no significant difference in p-Akt protein expression (*p* > 0.05) was observed in the PFC, as shown in Figures 3(d) and 3(e). In contrast, the p-Erk levels in the hippocampus and PFC were not significantly different among groups, as shown in Figures 3(c) and 3(f). This result suggests that the antidepressant effect of THSG involves the p-Akt pathway, and THSG treatment positively mediates p-Akt protein expression to exert neuroprotection in the CNS.

### 3.5. Effect of THSG Treatment on Neurogenesis

**Figure 4:**
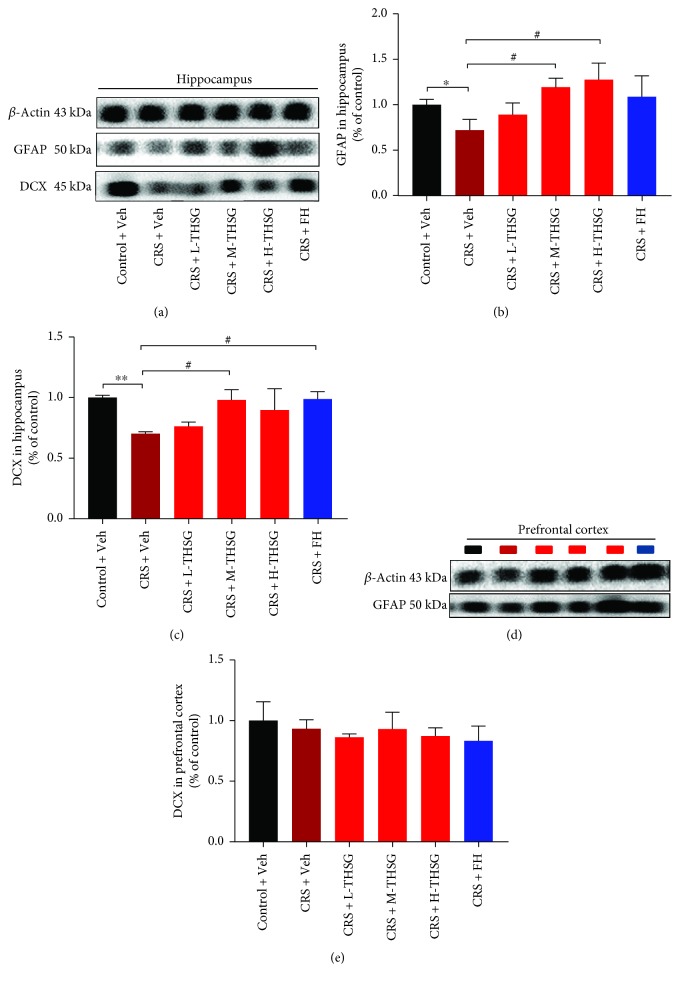
Effect of THSG treatment on GFAP and DCX expression in the hippocampus and PFC. The relative levels of GFAP and DCX protein expression in the hippocampus and prefrontal cortex are shown in (a, b, c). In addition, graph (d, e) shows the relative expression levels of GFAP in the PFC. The protein band densities were analyzed using the ImageJ software. Values are expressed as the mean ± SEM (*n* = 3). For statistical significance, ^∗^*p* < 0.05 and ^∗∗^*p* < 0.01 versus the control + Veh group; ^#^*p* < 0.05 versus the CRS + Veh group.

Restraint stress results in damage and cell loss in the CNS, which further induces cognition dysfunction [40]. The levels of glial fibrillary acidic protein (GFAP), a commonly applied astrocyte marker [41, 42], were used to estimate the state of astrocyte proliferation in CRS-induced mice. As shown in Figures 4(a) and 4(b), CRS exposure significantly downregulated GFAP protein expression in the hippocampus (*p* < 0.05) but not in the PFC (Figures 4(d) and 4(e), *p* > 0.05). As expected, THSG treatment reversed the decrease in GFAP protein expression in a dose-dependent manner (*p* < 0.05). Neurogenesis-related protein, DCX, an immature neuronal marker, was also detected. As shown in Figures 4(a) and 4(c), CRS stimulation reduced DCX protein expression in the mouse hippocampus(*p* < 0.01), indicative of reduced neurogenesis, and this change was notably inhibited by M-THSG treatment (40 mg/ml) (*p* < 0.05). However, differences between mice receiving L- and H-THSG were not apparent. This result confirmed that CRS stress induces cell damage in mice, and THSG may exert antidepressant effects by increasing neurogenesis and astrocyte proliferation.

## 4. Discussion

The pathological complexity of depression has undoubtedly led to inefficiencies in the development of antidepressant drugs over the past few decades [43]. In the present study, however, an effective antidepressant-like effect of THSG administration in an MDD mouse model induced by the CRS stimulus was verified, and the underlying mechanisms were evaluated by a series of methods. We found that THSG exerts its neuroprotective effect via various complex physical outlets related to the amelioration of oxidative stress in vivo, anti-inflammatory properties, and neurogenesis promotion. To our knowledge, this research is the first to demonstrate an antidepressant-like effect of THSG.

Chronic stressful life events represent some predisposing factors in the development of MDD [44, 45], and CRS is a method ubiquitously utilized to successfully establish MDD mouse model [46]. The mice exposed to the CRS paradigm exhibited many depressive characteristics, such as body weight loss and prolonged immobility times in the FST and TST. In addition, the autonomous motor abilities of MDD mice were not influenced in all the groups of our experiment, suggesting that the differences in immobility among groups are not caused by motor abilities. By contrast, the changes in mouse behavior induced by CRS were reversed and modified by THSG treatment. These behavioral indices were similar to those induced by the antidepressant FH.

Many studies have revealed that inflammation in the CNS is closely associated with the pathogenesis of depression [8]. Microglia, accounting for 10% of the hippocampus, olfactory telencephalon, basal ganglia, and substantia nigra [47], play an important role in CNS inflammation. High levels of proinflammatory factors, such as TNF-*α*, IL-1, and IL-6, are induced by dynamic microglial damaged neurons in MDD patients [48]. However, other studies reported no apparent association between higher proinflammatory factor levels and worsening depressive symptoms [49, 50]. Our data provide solid support for the former conclusion, and we further found that THSG also inhibits the upregulation of proinflammatory cytokines in MDD mice. These results indicated that THSG suppresses inflammatory responses in vivo.

The redox system is suggested to be activated not only during inflammation but also during the depressive process. Indeed, evidence has demonstrated that rats subjected to the chronic mild stress paradigm show elevated oxidative stress levels in the brain [51]. Moreover, inflammation can also result in the production of ROS [52]. As a consequence, the oxidative stress process arises from an imbalance between oxidant and antioxidant compounds in favor of excessive free radical generation. Within certain limits, ROS are balanced by antioxidant defenses, such as GSH-PX and T-AOC. However, excessive ROS production can ultimately lead to cellular damage via enzyme inactivation, lipid peroxidation, protein damage, and DNA modification [17, 53]. For example, a relatively lower total antioxidant status and higher MDA content were observed in the serum, hippocampus, and PFC of depressed rats [3, 54]. Our finding proved that MDA contents in the serum, hippocampus, and PFC are notably elevated in MDD mice and that THSG treatment can reverse this change. Notably, changes in the GSH-PX and T-AOC activities tended to parallel those in MDA content, which provided vigorous evidence supporting the hypothesis that the self-defensive system can resist oxidative damage to a certain degree.

Using neuroimaging paradigms, researchers discovered that PFC and hippocampal tissues in the brain play an important role in regulating mood responses [7]. Postmortem studies on depressed patients demonstrated reductions in the number and/or densities of glial and neuronal cells in several cortical areas [55]. Notably, prolonged CRS potentially leads to continuous neuronal changes in the brain, as reported by others [56]. A similar hypothesis proposes that stress alters glial cell function, consequently inducing neuronal atrophy, PFC impairment, and depression symptoms [57]. The expression of GFAP, regarded as a glial cell biomarker, was significantly downregulated in hippocampal tissues from depressed patients [10], and this change was reversed when the patients were treated [12]. This study also showed the effect of THSG treatment on upregulating GFAP protein expression in hippocampal tissue of MDD mice. The mRNA expression of DCX, a specific marker of newborn neurons, was increased during mouse hippocampus neurogenesis after short-term CRS exposure [58]. We observed that long-term CRS exposure markedly reduced DCX expression in the hippocampus, but DCX expression was recovered after THSG treatment. In addition, a previous study reported that proinflammatory cytokines can activate neuronal apoptotic pathways [59]. To further investigate the underlying mechanism, two classical neuroprotective pathways, the Akt and Erk signaling pathways, attracted our attention. We found that the Akt signaling pathway is probably linked to THSG neuroprotection via upregulating the expression of the p-Akt protein.

## 5. Conclusions

**Figure 5:**
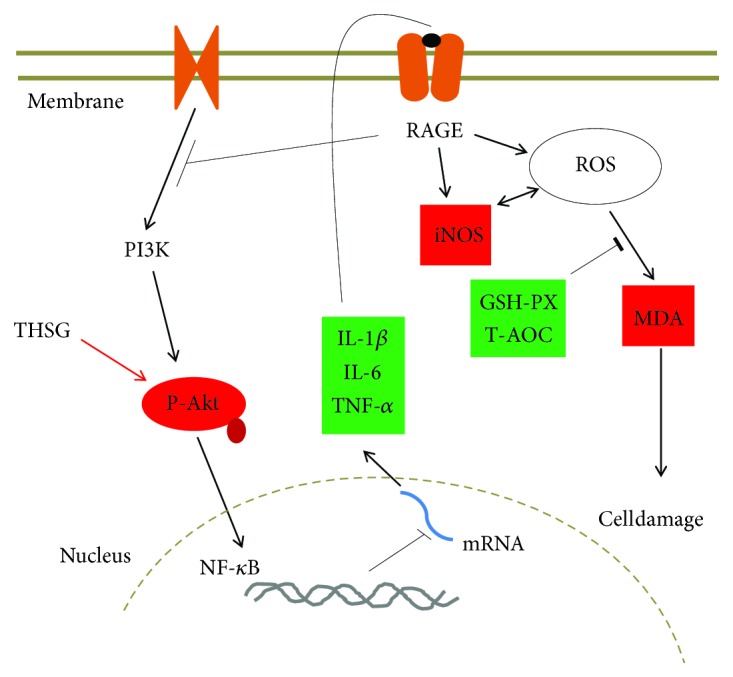
Potential mechanism of the THSG antidepressant effect.

In conclusion, this study has revealed for the first time that administering THSG can rescue the depressive-like behaviors and pathological features of CRS-induced model mice. Unlike the current single-target drugs, THSG is a multiple-target antidepressant drug that most likely prevents oxidative stress, exerts anti-inflammatory properties, and promotes neuroprotective signaling pathway (Figure 5). This conclusion is consistent with the view that targeting psychosis with multiple-target drugs rather than single-target drugs may be more effective [43].

## Figures and Tables

**Table 1:** The effect of THSG treatment on antioxidant ability in serum, hippocampus, and PFC tissues of mice.

Item	Sample	Groups (*n* = 9)					
		Control + Veh	CRS + Veh	CRS + L-THSG	CRS + M-THSG	CRS + H-THSG	CRS + FH
MDA	Serum (*μ*mol/l)	18.86 ± 1.75	23.50 ± 1.80^∗^	14.75 ± 1.32^###^	16.18 ± 1.39^##^	17.06 ± 1.25^##^	20.13 ± 1.43
	Hip (*μ*mol/mg)	0.65 ± 0.04	0.79 ± 0.03^∗∗^	0.58 ± 0.03^###^	0.47 ± 0.02^###^	0.64 ± 0.05^#^	0.66 ± 0.03^##^
	PFC (*μ*mol/mg)	0.84 ± 0.04	0.94 ± 0.04^∗^	0.88 ± 0.02	0.84 ± 0.04^#^	0.72 ± 0.03^###^	0.81 ± 0.05^#^

GSH-PX	Serum (U/ml)	905.1 ± 64.37	1089 ± 31.64^∗^	1128 ± 37.38	892.7 ± 50.81^##^	749.2 ± 78.05^##^	918.3 ± 36.97^##^
	Hip (U/mg)	169.1 ± 8.45	143.3 ± 6.73^∗^	145.7 ± 13.15	163.4 ± 13.01	176.3 ± 10.08^#^	178.6 ± 15.57^#^
	PFC (U/mg)	215.2 ± 15.37	320.3 ± 54.46^∗^	280.9 ± 42.50	200.5 ± 19.26^#^	249.5 ± 38.14	224.2 ± 17.13^#^

T-AOC	Serum (U/ml)	5.12 ± 1.05	6.11 ± 0.44	5.50 ± 0.52	5.34 ± 0.74	4.40 ± 0.50^#^	5.56 ± 0.58
	Hip (U/mg)	0.188 ± 0.039	0.258 ± 0.09^∗^	0.222 ± 0.017	0.161 ± 0.012^##^	0.205 ± 0.039	0.201 ± 0.029^#^
	PFC (U/mg)	0.24 ± 0.047	0.26 ± 0.046	0.16 ± 0.014^#^	0.10 ± 0.012^##^	0.19 ± 0.052	0.15 ± 0.049^#^

Note: MDA: malondialdehyde; GSH-PX: glutathione peroxidase; T-AOC: total antioxidant capacity; Hip: hippocampus; PFC: prefrontal cortex. Effect of THSG treatment on the antioxidant abilities in mouse serum, hippocampal tissue, and cortex tissue. Peripheral serum samples were collected from the retroorbital vessel after drug administration, and the hippocampal and prefrontal cortex tissues were dissected after the mice were sacrificed. The MDA content and GSHPX and T-AOC activities in serum are expressed as *μ*mol/l, U/ml, and U/ml, respectively, while those values in the hippocampal and prefrontal cortex tissues are expressed as *μ*mol/mg, U/mg, and U/mg, respectively. Values are expressed as the mean ± SEM, with 10 mice in the control + Veh group and 9 mice in the other groups. For statistical significance, ^∗^*p* < 0.05 and ^∗∗^*p* < 0.01 versus the control + Veh group; ^#^*p* < 0.05, ^##^*p* < 0.01, and ^###^*p* < 0.001 versus the CRS + Veh group.

## Data Availability

The data used to support the findings of this study are available from the corresponding author upon request.
